# Expanding CRISPR repertoire using CjCas9 as a smaller editing tool

**DOI:** 10.1016/j.omtn.2022.09.013

**Published:** 2022-09-30

**Authors:** Christopher Francis, Mansoor Amiji

**Affiliations:** 1 Department of Pharmaceutical Sciences, School of Pharmacy and Pharmaceutical Sciences, Northeastern University, Boston, MA 02115, USA; 2 Pfizer, Inc, Worldwide Research & Development, Medicinal Sciences, Biomedicine Design, Andover, MA 01810, USA; 3 Department of Chemical Engineering, College of Engineering, Northeastern University, Boston, MA 02115, USA

## Abstract

The field of gene editing continues to expand significantly and is entering a time of unprecedented utility. Academia and industry look to conquer genetic-based disease with viral and non-viral-delivered CRISPR-Cas9. The most widely used Cas9 protein is derived from *Streptococcus pyrogenes* (SpCas9), which lends itself to being too large for AAV viral delivery. Cas9 orthologue proteins have diverse size and dependent on bacteria of origin. This diversity has given rise to Cas9 proteins smaller in size while maintaining gene editing abilities. In this article, authors have focused on the use of CjCas9, whose smaller size allows for packaging in AAV and maintains high on-target gene editing. The locus APOC3 was identified for eventual targeting/integration in humans where cardioprotective properties are predicted. To confirm *in vivo* targeting of this locus, a humanized mouse model was developed due to the absence of the APOC3 locus in mice. These studies looked to answer long-standing questions on integrated gene stability, promoter/low gene integration, and the duration of therapeutic efficacy of the integrated gene.

## Teaser

The field of gene editing continues to expand significantly and is entering a time of unprecedented utility. Academia and industry look to conquer genetic-based disease with viral and non-viral-delivered CRISPR-Cas9. The most widely used Cas9 protein is derived from *Streptococcus pyrogenes* (SpCas9), which lends itself to being too large for AAV viral delivery. Cas9 orthologue proteins have been found to have diverse size and dependent on bacteria of origin. This diversity has given rise to Cas9 proteins smaller in size while maintaining gene editing abilities. A recent study focused on the use of CjCas9, whose smaller size allows for packaging in AAV and maintains high on-target gene editing. The locus APOC3 was identified for eventual targeting/integration in humans where cardioprotective properties are predicted. To confirm *in vivo* targeting of this locus, a humanized mouse model was developed due to the absence of the APOC3 locus in mice. These studies looked to answer long-standing questions on integrated gene stability, promoter/low gene integration, and the duration of therapeutic efficacy of the integrated gene.

## Main text

CRISPR-Cas9 gene editing has shown wide use with delivery using viral and non-viral-based delivery methods. SpCas9 has seen the most dominant use but limited when it comes to adeno-associated virus (AAV)-based gene therapy. AAV-delivered gene therapy is under way in clinical trials, with several therapies on the verge of commercialization. The ability to couple AAV with CRISPR-Cas9 could mark the next era in gene therapy. One limitation with SpCas9 and AAV is the finite space for genome packing, but smaller Cas9 proteins could expand its implementation. Orthologues of the Cas9 protein are diverse, with several Cas9 proteins falling in the size category that allows for compatibility with AAV (Table 1). Cas9 protein derived from *Campylobacter jejuni* (CjCas9) possesses this size requirement^1^, along with an increase in PAM sequence recognition offering enhanced on-target gene editing. The cleavage of the genome relies on the Cas9 nuclease domains driven by protein/DNA interaction, while the PAM sequence plays an integral role in recognition of the target DNA strand. An NGG PAM sequence is used by SpCas9, while CjCas9 uses an expanded NNNNRYAC or NNNNACAC PAM making sequence recognition more precise. This increase in specificity and low off-target cleavage may aid in concerns related to the persistent expression of AAV *in vivo.*

**Table 1:** A limited number of Type-II Cas9 orthologues shown that are compatible with AAV Packaging^1^

Cas9 orthologue	Size (base pairs)	PAM
SpCas9	4,200	NGG
CjCas9	2,952	NNNNACAC/NNNNRYAC
SaCas9	3,159	NNNRRT
Nme2Cas9	3,246	NNNNCC

In this study, the authors set out to identify targets *in vitro* and used those targets for *in vivo* studies to modify hemophilia disease. Researchers identified target loci in HEPG2 cells with high promoter expression and Cas9 targeting. *In silico* digenome-sequencing and targeting deep sequencing demonstrated high on-target with little to no off-target for the loci APOC3 (apolipoprotein C3)and HP (haptoglobin) *in vitro*. Advantages in the APOC3 locus offer: (1) high on-target Cas9 specificity, (2) could provide cardioprotective benefits in humans as a site for payload integration, and (3) it contains a strong promoter for payload expression. CjCas9 targeted the APOC3 locus, and with the delivery of GFP payload, sustained integration/expression was observed. The GFP payload was then replaced with Factor IX (FIX) with integration relying on either homologous recombination (HR) or homology-independent target integration (HITI). Both methods showed increases in relative FIX mRNA expression *in vitro* at the APOC3 target site. Researchers developed a humanized mouse model to ascertain APOC3 locus targeting *in vivo*. Early *in vivo* work with this model used AAV8 packaged CjCas9 and single guide RNA (sgRNA) targeting APOC3. Locus targeting was dose-dependent, and specificity was validated as *in silico* predicted off-targets were not observed. The integration of payload tested the use of either homology arm (HA)-driven HR or HITI/AAV trap independent of genome homology integration. The highest expression of FIX came from HA/HR driven FIX payload integration. Reduced expression was seen with payload integration that was independent of sequence homology. Albeit lower FIX expression, the bidirectional FIX payload/AAV trap showed 250 ng/mL followed by HITI payload at 50 ng/mL over 22 weeks. This work’s final study expanded to include the Hemophilic APOC3+ mouse model to test therapeutic relevance. The co-dosing of AAV CjCas9/sgRNA and a bidirectional FIX payload were tested in this model. Treatment with AAV8 was confined to the liver, with other organs showing little to no editing or off-target editing. The co-delivery of two AAVs showed relevant FIX expression, demonstrated with decreases in clotting times using the activated partial thromboplastin time assay.

The authors utilize proven AAV facilitated gene editing and overcame the limitations associated with SpCas9 viral packing size using the CjCas9 protein. The notion of payload gene placement to an area of the genome containing an endogenous strong promoter for the purpose of high gene expression is innovative. *Lee* *et al*^2^*.* highlight even with expected low integration efficiency this can be overcome by a strong promoter for therapeutic levels of gene expression. The targeting of loci *in vitro* into relevant *in vivo* models typically focus on preexisting loci, but here the APOC3 gene target could provide benefit in humans but not found in mice. The authors developed a mouse model that contains the APOC3 locus, a target for clinical purposes in humans. The APOC3/FIX mouse allowed to not only test the type of payload integration being used but also the duration of the exogeneous FIX gene expression. In a clinical setting, this method could have implications not only for the disease by knocking out a gene that has negative effects, but with the introduction of a gene that has therapeutic benefit. AAV gene therapy is expanding as the Cas9 toolbox increases with the introduction of Cas9 orthologues. The reduced size found in several Cas9 proteins will lend utility for gene editing in AAV. As with SpCas9, the crystal structure for CjCas9 is available and allows for increased knowledge of specific interactions between the PAM and target/non-target DNA strands for this orthologue. Similarities between SpCas9 and CjCas9 are seen with allosteric communication between the two nuclease domains, RuvC and HCH.^3^ Information revealed by this structure and future research with the CjCas9 protein could elucidate details around protein-DNA interactions and identify areas necessary and those not required for gene editing. This may allow for further miniaturization of Cas9 orthologues increasing available space in the AAV virion for additional sequences: gene payload or accessories sequences. The ability to stay within the confines of a single AAV containing Cas9/sgRNA and a donor gene for integration could help shape the future of viral-based gene therapy.
